# 2,7-Dimethyl-1,3-thia­zolo[4,5-*d*]pyridazin-4(5*H*)-one

**DOI:** 10.1107/S1600536811034192

**Published:** 2011-08-27

**Authors:** Abdulrahman O. Al-Youbi, Abdullah M. Asiri, Hassan M. Faidallah, Seik Weng Ng

**Affiliations:** a Chemistry Department, Faculty of Science, King Abdulaziz University, PO Box 80203, Jeddah, Saudi Arabia; b Department of Chemistry, University of Malaya, 50603 Kuala Lumpur, Malaysia

## Abstract

The nine-membered fused-ring system of the title pyridazine derivative, C_7_H_7_N_3_OS, is almost planar (r.m.s. deviation 0.012 Å). In the crystal, the amino H atom forms a hydrogen bond to the ketonic O atom of a neighboring mol­ecule to generate a centrosymmetric dimer.

## Related literature

For a related structure, see: Abdel-Aziz *et al.* (2010 ▶). For the biological activity of the class of pyridazines, see: Faid-Allah *et al.* (2011 ▶); Makki & Faid-Allah (1996 ▶).
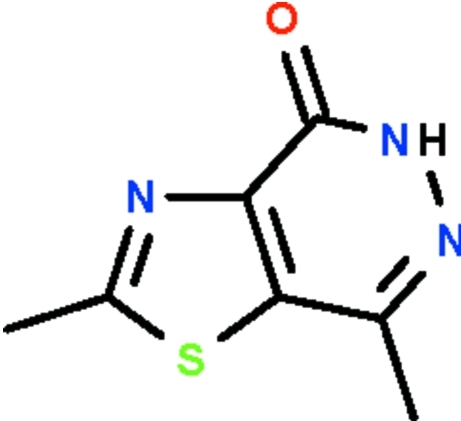


## Experimental

### 

#### Crystal data

C_7_H_7_N_3_OS
*M* *_r_* = 181.22
Triclinic, 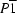

*a* = 6.9262 (4) Å
*b* = 7.0540 (4) Å
*c* = 8.8079 (6) Å
α = 71.002 (6)°
β = 75.845 (5)°
γ = 85.570 (5)°
*V* = 394.54 (4) Å^3^
*Z* = 2
Cu *K*α radiation
μ = 3.26 mm^−1^
*T* = 100 K
0.30 × 0.25 × 0.20 mm

#### Data collection

Agilent Technologies SuperNova Dual diffractometer with Atlas detector
Absorption correction: multi-scan (*CrysAlis PRO*; Agilent, 2010 ▶) *T*_min_ = 0.442, *T*_max_ = 0.562
2363 measured reflections
1539 independent reflections
1523 reflections with *I* > 2σ(*I*)
*R*_int_ = 0.012

#### Refinement

*R*[*F* ^2^ > 2σ(*F* ^2^)] = 0.029
*wR*(*F* ^2^) = 0.080
*S* = 1.05
1539 reflections
115 parameters
H atoms treated by a mixture of independent and constrained refinement
Δρ_max_ = 0.41 e Å^−3^
Δρ_min_ = −0.30 e Å^−3^

### 

Data collection: *CrysAlis PRO* (Agilent, 2010 ▶); cell refinement: *CrysAlis PRO*; data reduction: *CrysAlis PRO*; program(s) used to solve structure: *SHELXS97* (Sheldrick, 2008 ▶); program(s) used to refine structure: *SHELXL97* (Sheldrick, 2008 ▶); molecular graphics: *X-SEED* (Barbour, 2001 ▶); software used to prepare material for publication: *publCIF* (Westrip, 2010 ▶).

## Supplementary Material

Crystal structure: contains datablock(s) global, I. DOI: 10.1107/S1600536811034192/xu5288sup1.cif

Structure factors: contains datablock(s) I. DOI: 10.1107/S1600536811034192/xu5288Isup2.hkl

Supplementary material file. DOI: 10.1107/S1600536811034192/xu5288Isup3.cml

Additional supplementary materials:  crystallographic information; 3D view; checkCIF report

## Figures and Tables

**Table 1:** Hydrogen-bond geometry (Å, °)

*D*—H⋯*A*	*D*—H	H⋯*A*	*D*⋯*A*	*D*—H⋯*A*
N2—H2⋯O1^i^	0.88 (2)	1.97 (2)	2.845 (2)	173 (2)

Symmetry code: (i) 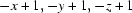.

**Fig. 1.**
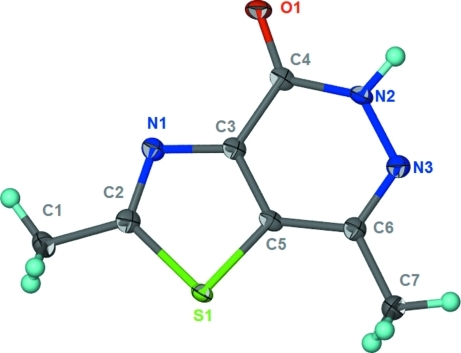
Thermal ellipsoid plot (Barbour, 2001) of C7H7N3OS at the 70% probability level; hydrogen atoms are drawn as spheres of arbitrary radius.

## supplementary crystallographic information

### Comment

We have reported the synthesis of some pyridazines, which exhibit biological activity (Faid-Allah *et al.*, 2011; Makki & Faid-Allah, 1996). There are few crystal structure reports of such systems; recently, we reported the crystal structure of 3-methyl-2-(4-methyl)-2*H*-pyrazolo[3,4-*d*]pyridazin-5-ium thiocyanate, a salt (Abdel-Aziz *et al.*, 2010). The nine-membered fused-ring system of C_7_H_7_N_3_OS (Scheme I) is planar (Fig. 1). The amino group forms a hydrogen bond to the ketonic O atom of a neigboring molecule to form a dimer (Table 1).

### Experimental

A solution of ethyl 5-acetyl-3-methylisoxazole-4-carboxylate (2.10 g, 10 mmol) in ethanol (25 ml) was refluxed with hydrazine hydrate (0.50 g, 10 mmol) for 2 h. The pyridazine which separated after concentration of the reaction mixture was filtered off, washed with ethanol and recrystallized from the same solvent to give colorless prisms in 90% yield, mp 527 K.

### Refinement

Carbon-bound H-atoms were placed in calculated positions [C—H 0.95 to 0.98 Å, *U*_iso_(H) 1.2 to 1.5*U*_eq_(C)] and were included in the refinement in the riding model approximation.

The amino H-atom was located in a difference Fourier map, and were refined freely.

Omitted were (4 0 4), (1 0 1) and (-7 - 2 1).

### Crystal data

**Table:**

C_7_H_7_N_3_OS	*Z* = 2
*M**_r_* = 181.22	*F*(000) = 188
Triclinic, *P*1	*D*_x_ = 1.525 Mg m^−^^3^
Hall symbol: -P 1	Cu *K*α radiation, λ = 1.54184 Å
*a* = 6.9262 (4) Å	Cell parameters from 1965 reflections
*b* = 7.0540 (4) Å	θ = 6.6–74.2°
*c* = 8.8079 (6) Å	µ = 3.26 mm^−^^1^
α = 71.002 (6)°	*T* = 100 K
β = 75.845 (5)°	Prism, colorless
γ = 85.570 (5)°	0.30 × 0.25 × 0.20 mm
*V* = 394.54 (4) Å^3^	

### Data collection

**Table:**

Agilent Technologies SuperNova Dual diffractometer with Atlas detector	1539 independent reflections
Radiation source: SuperNova (Cu) X-ray Source	1523 reflections with *I* > 2σ(*I*)
Mirror	*R*_int_ = 0.012
Detector resolution: 10.4041 pixels mm^-1^	θ_max_ = 74.4°, θ_min_ = 6.6°
ω scan	*h* = −8→7
Absorption correction: multi-scan (*CrysAlis PRO*; Agilent, 2010)	*k* = −8→4
*T*_min_ = 0.442, *T*_max_ = 0.562	*l* = −10→10
2363 measured reflections	

### Refinement

**Table:**

Refinement on *F*^2^	Primary atom site location: structure-invariant direct methods
Least-squares matrix: full	Secondary atom site location: difference Fourier map
*R*[*F*^2^ > 2σ(*F*^2^)] = 0.029	Hydrogen site location: inferred from neighbouring sites
*wR*(*F*^2^) = 0.080	H atoms treated by a mixture of independent and constrained refinement
*S* = 1.05	*w* = 1/[σ^2^(*F*_o_^2^) + (0.0494*P*)^2^ + 0.260*P*] where *P* = (*F*_o_^2^ + 2*F*_c_^2^)/3
1539 reflections	(Δ/σ)_max_ = 0.001
115 parameters	Δρ_max_ = 0.41 e Å^−^^3^
0 restraints	Δρ_min_ = −0.30 e Å^−^^3^

### Fractional atomic coordinates and isotropic or equivalent isotropic displacement parameters (Å^2^)

**Table:**

	*x*	*y*	*z*	*U*_iso_*/*U*_eq_	
S1	1.29210 (5)	0.13257 (5)	0.50269 (4)	0.01037 (14)	
O1	0.59921 (15)	0.34439 (15)	0.67118 (13)	0.0136 (2)	
N1	0.97795 (18)	0.13725 (17)	0.73210 (15)	0.0114 (3)	
N2	0.74300 (18)	0.42456 (18)	0.39677 (15)	0.0112 (3)	
H2	0.636 (3)	0.490 (3)	0.371 (3)	0.027 (5)*	
N3	0.88967 (18)	0.43408 (18)	0.25907 (15)	0.0121 (3)	
C1	1.2623 (2)	−0.0391 (2)	0.84407 (19)	0.0157 (3)	
H1A	1.1669	−0.0660	0.9512	0.024*	
H1B	1.3732	0.0410	0.8415	0.024*	
H1C	1.3131	−0.1663	0.8277	0.024*	
C2	1.1611 (2)	0.0736 (2)	0.70979 (18)	0.0118 (3)	
C3	0.9338 (2)	0.2388 (2)	0.58191 (17)	0.0103 (3)	
C4	0.7459 (2)	0.3356 (2)	0.56003 (18)	0.0107 (3)	
C5	1.0839 (2)	0.2502 (2)	0.44375 (18)	0.0101 (3)	
C6	1.0596 (2)	0.3495 (2)	0.27985 (18)	0.0111 (3)	
C7	1.2245 (2)	0.3608 (2)	0.13145 (18)	0.0167 (3)	
H7A	1.1756	0.4244	0.0312	0.025*	
H7B	1.2713	0.2252	0.1345	0.025*	
H7C	1.3346	0.4401	0.1314	0.025*	

### Atomic displacement parameters (Å^2^)

**Table:**

	*U*^11^	*U*^22^	*U*^33^	*U*^12^	*U*^13^	*U*^23^
S1	0.0081 (2)	0.0092 (2)	0.0123 (2)	0.00189 (13)	−0.00196 (13)	−0.00205 (14)
O1	0.0089 (5)	0.0137 (5)	0.0156 (5)	0.0007 (4)	0.0001 (4)	−0.0034 (4)
N1	0.0113 (6)	0.0082 (6)	0.0136 (6)	−0.0004 (4)	−0.0028 (5)	−0.0019 (5)
N2	0.0081 (6)	0.0104 (6)	0.0140 (6)	0.0020 (5)	−0.0022 (5)	−0.0032 (5)
N3	0.0120 (6)	0.0103 (6)	0.0130 (6)	0.0000 (5)	−0.0018 (5)	−0.0029 (5)
C1	0.0147 (7)	0.0150 (7)	0.0155 (7)	0.0025 (6)	−0.0051 (6)	−0.0015 (6)
C2	0.0124 (7)	0.0076 (6)	0.0142 (7)	−0.0016 (5)	−0.0020 (5)	−0.0021 (5)
C3	0.0103 (7)	0.0057 (6)	0.0137 (7)	−0.0016 (5)	−0.0019 (5)	−0.0016 (5)
C4	0.0103 (7)	0.0057 (6)	0.0157 (7)	−0.0021 (5)	−0.0030 (5)	−0.0025 (5)
C5	0.0095 (6)	0.0060 (6)	0.0151 (7)	0.0002 (5)	−0.0034 (5)	−0.0034 (5)
C6	0.0122 (7)	0.0078 (6)	0.0127 (7)	−0.0011 (5)	−0.0026 (5)	−0.0022 (5)
C7	0.0151 (7)	0.0191 (8)	0.0127 (7)	0.0022 (6)	−0.0017 (6)	−0.0022 (6)

### Geometric parameters (Å, °)

**Table:**

S1—C5	1.7141 (14)	C1—H1A	0.9800
S1—C2	1.7546 (15)	C1—H1B	0.9800
O1—C4	1.2404 (18)	C1—H1C	0.9800
N1—C2	1.3025 (19)	C3—C5	1.378 (2)
N1—C3	1.3797 (18)	C3—C4	1.4480 (19)
N2—N3	1.3647 (17)	C5—C6	1.430 (2)
N2—C4	1.3736 (19)	C6—C7	1.4953 (19)
N2—H2	0.88 (2)	C7—H7A	0.9800
N3—C6	1.3037 (19)	C7—H7B	0.9800
C1—C2	1.493 (2)	C7—H7C	0.9800
			
C5—S1—C2	89.25 (7)	N1—C3—C4	125.15 (13)
C2—N1—C3	110.07 (12)	O1—C4—N2	120.86 (13)
N3—N2—C4	129.17 (12)	O1—C4—C3	126.43 (13)
N3—N2—H2	111.4 (14)	N2—C4—C3	112.70 (12)
C4—N2—H2	119.3 (14)	C3—C5—C6	122.57 (13)
C6—N3—N2	117.76 (12)	C3—C5—S1	109.49 (11)
C2—C1—H1A	109.5	C6—C5—S1	127.94 (11)
C2—C1—H1B	109.5	N3—C6—C5	119.21 (13)
H1A—C1—H1B	109.5	N3—C6—C7	119.01 (13)
C2—C1—H1C	109.5	C5—C6—C7	121.77 (13)
H1A—C1—H1C	109.5	C6—C7—H7A	109.5
H1B—C1—H1C	109.5	C6—C7—H7B	109.5
N1—C2—C1	125.25 (13)	H7A—C7—H7B	109.5
N1—C2—S1	114.91 (11)	C6—C7—H7C	109.5
C1—C2—S1	119.83 (11)	H7A—C7—H7C	109.5
C5—C3—N1	116.28 (12)	H7B—C7—H7C	109.5
C5—C3—C4	118.56 (13)		
			
C4—N2—N3—C6	−0.4 (2)	N1—C3—C5—C6	179.58 (12)
C3—N1—C2—C1	178.83 (13)	C4—C3—C5—C6	−1.6 (2)
C3—N1—C2—S1	−0.33 (15)	N1—C3—C5—S1	−0.99 (16)
C5—S1—C2—N1	−0.18 (11)	C4—C3—C5—S1	177.81 (10)
C5—S1—C2—C1	−179.39 (12)	C2—S1—C5—C3	0.63 (10)
C2—N1—C3—C5	0.86 (17)	C2—S1—C5—C6	−179.99 (13)
C2—N1—C3—C4	−177.85 (12)	N2—N3—C6—C5	0.55 (19)
N3—N2—C4—O1	178.56 (12)	N2—N3—C6—C7	−178.84 (12)
N3—N2—C4—C3	−0.7 (2)	C3—C5—C6—N3	0.5 (2)
C5—C3—C4—O1	−177.59 (13)	S1—C5—C6—N3	−178.85 (10)
N1—C3—C4—O1	1.1 (2)	C3—C5—C6—C7	179.84 (13)
C5—C3—C4—N2	1.63 (18)	S1—C5—C6—C7	0.5 (2)
N1—C3—C4—N2	−179.69 (12)		

### Hydrogen-bond geometry (Å, °)

**Table:**

*D*—H···*A*	*D*—H	H···*A*	*D*···*A*	*D*—H···*A*
N2—H2···O1^i^	0.88 (2)	1.97 (2)	2.845 (2)	173 (2)

Symmetry codes: (i) −*x*+1, −*y*+1, −*z*+1.
